# Tako Tsubo and Treatment of Comorbid Depression: A Reflection is Needed - Author's reply

**DOI:** 10.2174/1745017901915010003

**Published:** 2019-01-23

**Authors:** Antonio Preti, Federica. Sancassiani

**Affiliations:** Department of Medical Sciences and Public Health, University of Cagliari, Cagliari, Italy

Finsterer and Stollberger [1] raised several interesting points concerning the results of our research on Tako Tsubo Syndrome (TTS) [2]. With co-authors, we have already explained why we do not agree with most of them [3]. However, one point seems to us worthy of further discussion, that is the possible role of antidepressants in the etiology of TTS. Contrarily to what Finsterer and Stollberger [1] have intended, we did not state anything about the links between use of antidepressants and TTS. We have investigated the link since antidepressants are the most prescribed drugs in patients diagnosed with the Major Depressive Disorder (MDD). We found people with TTS had a greater chance of using antidepressants at the epoch of the TTS, but the odds failed to reach the statistically significant level after correction for comorbid MDD. As a matter of fact, several case reports have been reported of an association of TTS with antidepressant use [4, 5]. Although a case report is not scientific evidence, the set of all case reports had to be interpreted in this framework. Moreover, immediately after the publication of our study, a review came out supporting the association between TTS and mood disorders, with a role for a link between TTS and antidepressants [5]. Overall, we think that the doubts of the commentary authors as far as the role of the use of antidepressants in TTS seem outdated.

Nevertheless, we concede to Finsterer and Stollberger that the data in so far is strongly suggestive but not yet exhaustive. However, we think that it is time to definitively clear whether the risk exists, what is the pathogenesis, and which antidepressants are more likely implicated.

In fact, there are some antidepressants which more than others can potentially increase catecholamine’s level. We refer to SNRIs, for which a possible risk of inducing arterial hypertension has also been reported [6], and, as well, an increased risk of arrhythmia among elderly with previous cardiovascular events [7]. The prescription of these antidepressants in patients at risk of TTS should be carefully weighed against the risk of inducing a surge in the catecholamine tone and strongly monitored. It should be noted that these antidepressants are of the second choice in most of the guidelines with respect to SSRIs, acting only on serotonin, nevertheless, SNRIs have had a notable increase in the recent past. In patients with a potential risk of TTS, the use of SSRIs (however of the first choice) would be preferable because they are less able to increase catecholamine tone centrally and peripherally. Electroconvulsive Therapy (ECT) may be an alternative since the existence of acute cardiovascular risks has always been denied for ECT.

However, several case reports had indicated a link of ECT with TTS, thus the use of ECT in patients at risk of TTS requires more detailed monitoring from now on.

In conclusion, antidepressants might have a role in TTS, and prescribing an antidepressant to treat MDD in patients at risk of TTS is not without consequences.
